# The German version of the Posttraumatic Stress Disorder Checklist for DSM-5 (PCL-5): psychometric properties and diagnostic utility

**DOI:** 10.1186/s12888-017-1541-6

**Published:** 2017-11-28

**Authors:** Antje Krüger-Gottschalk, Christine Knaevelsrud, Heinrich Rau, Anne Dyer, Ingo Schäfer, Julia Schellong, Thomas Ehring

**Affiliations:** 1 Institute of Psychology, University of Münster, Fliednerstrasse 21, 48149 Münster, Germany; 2 Department of Clinical Psychology and Psychotherapy, Free University Berlin, 14195 Berlin, Germany; 3 Psychotrauma Centre, German Armed Forces Hospital Berlin, 10179 Berlin, Germany; 4 Central Institute of Mental Health, Medical Faculty Mannheim/Heidelberg University, 68159 Mannheim, Germany; 5 Centre for Interdisciplinary Addiction Research, University of Hamburg, 20246 Hamburg, Germany; 6 Department of Psychiatry and Psychotherapy, University Medical Centre Hamburg-Eppendorf, 20246 Hamburg, Germany; 7 Department of Psychotherapy and Psychosomatic Medicine, Technical University Dresden, 01307 Dresden, Germany; 8 Department of Psychology, LMU, 80802 Munich, Germany

**Keywords:** Posttraumatic stress disorder, DSM-5, PCL-5, Self-report questionnaire

## Abstract

**Background:**

The Posttraumatic Stress Disorder (PTSD) Checklist (PCL, now PCL-5) has recently been revised to reflect the new diagnostic criteria of the disorder.

**Methods:**

A clinical sample of trauma-exposed individuals (*N* = 352) was assessed with the Clinician Administered PTSD Scale for DSM-5 (CAPS-5) and the PCL-5. Internal consistencies and test-retest reliability were computed. To investigate diagnostic accuracy, we calculated receiver operating curves. Confirmatory factor analyses (CFA) were performed to analyze the structural validity.

**Results:**

Results showed high internal consistency (α = .95), high test-retest reliability (*r* = .91) and a high correlation with the total severity score of the CAPS-5, *r* = .77. In addition, the recommended cutoff of 33 on the PCL-5 showed high diagnostic accuracy when compared to the diagnosis established by the CAPS-5. CFAs comparing the DSM-5 model with alternative models (the three-factor solution, the dysphoria, anhedonia, externalizing behavior and hybrid model) to account for the structural validity of the PCL-5 remained inconclusive.

**Conclusions:**

Overall, the findings show that the German PCL-5 is a reliable instrument with good diagnostic accuracy. However, more research evaluating the underlying factor structure is needed.

**Electronic supplementary material:**

The online version of this article (10.1186/s12888-017-1541-6) contains supplementary material, which is available to authorized users.

## Background

The diagnosis of posttraumatic stress disorder (PTSD) has undergone major changes with the transition from the Diagnostic and Statistical Manual of Mental Disorders, 4th edition (DSM-IV) to DSM-5 [1]. These include an expansion from three to four symptom clusters, the introduction of three new symptoms, and the revision of some already existing symptoms (for an overview, see [2]). As the transition from DSM-IV to DSM-5 included substantial changes to the definition of PTSD, existing questionnaires used to assess PTSD needed to be revised by adding new items for symptoms added to the PTSD diagnosis, removing items that are no longer part of the DSM-5 definition, and rephrasing some items.

The Posttraumatic Stress Disorder Checklist (PCL; [3]) is one of the most widely used self-report questionnaire to asses PTSD and has now been revised to correspond to the new DSM-5 criteria of PTSD (PCL-5; [4]). Changes between the PCL for DSM-IV and the PCL-5 include (a) adding three new items to assess the new PTSD symptoms blame, negative emotions, and reckless or self-destructive behavior, (b) changing the rating from a 1-5 scale to a 0-4 scale, (c) rewording of existing items to reflect the DSM-5 criteria, and (d) having only one PCL version instead of three versions for military members, civilians and specific events.

### Psychometric properties of the PCL-5

To our knowledge, four published studies to date have validated the new PCL-5; three were conducted in military or veteran samples ([5, 6, 7]; note that in reference 7 a preliminary version of the PCL-5 was used) and one in a college student sample [8]. In addition to the original English PCL-5, a Swedish version [9] and a Chinese version [10, 11] have also been examined.

Results show high internal consistencies for the total scale (α = .90 - .96) as well as the four subscales (intrusions: α = .77 - .92; avoidance: α = .74 – .92; negative alterations in cognitions and mood: α = .78 - .89; hyperarousal: α = .75 - .84) [5, 6, 8, 9]. In addition, high re-test reliability has been found in three studies (*r =* .66-.84) [6, 8, 9].

There is consistent evidence for high concurrent validity of the PCL-5 in the sense of high correlations with other symptom measures of PTSD (*r* = .84 - .87) [5–8]. Furthermore, some evidence supporting discriminant validity of the questionnaire was found in that the PCL-5 score is more strongly correlated with measures of related constructs (e.g., other measures of PTSD, depression, anxiety symptoms) than those of unrelated constructs (e.g., personality features, alcohol abuse, psychopathy) [5, 6, 8]. In sum, there is emerging data showing good psychometric properties for the PCL-5.

### Diagnostic utility of the PCL-5

According to its developers, one of the purposes of the PCL-5 is to screen individuals for PTSD and make a provisional PTSD diagnosis.^1^ In order to test the diagnostic utility of the PCL-5 as a screening instrument, it appears necessary to compare it to a gold standard structured clinical interview, such as the Clinician-Administered PTSD Scale for DSM-5 (CAPS-5) [12]. To our knowledge, this has only been reported in two studies to date, namely Marmar et al. [7] with a preliminary version of the PCL-5 and in Bovin et al. [6] where the PCL-5 was evaluated against a CAPS-5 diagnosis of PTSD. Results showed that cutoff scores of 31-33 on the PCL-5 showed the best diagnostic utility in predicting CAPS diagnoses, with no difference between the three scores (sensitivity = .88, specificity = .69, overall efficiency = .80) [6]. This is in line with the cutoff of 33 suggested by the developers of the PCL-5^1^. An alternative scoring method for the PCL-5 is treating each item rated as at least 2 (moderately) as a symptom endorsed and then following the DSM-5 diagnostic rule to establish a provisional PTSD diagnosis. When applying this rule to the PCL-5, Bovin and colleagues [6] also found good diagnostic agreement with the CAPS-5 (sensitivity = .81, specificity = .71, overall efficiency = .78). Although this recent study suggests that the PCL-5 possesses adequate diagnostic utility to be used as screening instrument for PTSD, clearly more research is needed comparing the PCL-5 to the gold standard diagnosis established from a structured clinical interview in additional samples.

### Structural validity of the PCL-5

Based on confirmatory factor analytic (CFA) studies on the structure of posttraumatic stress disorder (PTSD) criteria [13, 14], the DSM-5 revised the diagnostic structure of PTSD from a three-factor-model with 17 symptoms to a four-factor-model comprising 20 symptoms. Criterion B (reliving) underwent minor changes, criterion C was separated into two criteria (active avoidance and negative cognitions/moods) and in criterion D (alterations in arousal/ reactivity) a specification for anger expression and an additional symptom of reckless or self-destructive behaviour was included.

To our knowledge, seven published studies to date have tested whether this 4-factor structure can be supported when applying CFA to the PCL-5 [6, 8, 10, 15–18].^2^ In most studies, the DSM-5 model showed poor fit with the data. Even in the minority of studies where acceptable to good fit was found for the DSM-5 model [5, 6, 16, 19], there were other models that showed significantly better fit. In sum, support for the four-factor DSM-5 model when applied to the PCL-5 is poor.

Five alternative models have recently received most attention in the empirical literature (see also Table 1 for an overview of the different models). This includes (a) the *Dysphoria Model* [20] that was modified from the original model due to different and additional symptoms in the DSM-5 and comprises the four factors re-experiencing, avoidance, dysphoria, hyperarousal, (b) the five-factor *Dysphoric Arousal Model* [21], also modified due to the DSM-5 changes, separating hyperarousal into the two distinct clusters of dysphoric arousal and anxious arousal, (c) the six-factor *Anhedonia Model* [10] extending the Dysphoric Arousal Model by separating the Negative Alterations in Cognition and Mood factor into two distinct factors representing changes in negative vs. positive affect, (d) the six-factor *Externalizing Behavior Model* [19] also extending the Dysphoric Arousal Model by separating the Dysphoric Arousal factor into two separate factors of External Arousal vs. Externalizing Behavior, and (e) a seven-factor *Hybrid Model* [15] combining the Anhedonia and Externalizing Behavior Models described above.

**Table 1:** Item mapping for the alternative latent structure models

	DSM-5 model	Dysphoria model	Dysphoric Arousal model	Anhedonia model	Externalizing behavior model	Hybrid model	Three-Factor model
1. Intrusive thoughts	R	R	R	R	R	R	Factor 1
2. Nightmares	R	R	R	R	R	R	Factor 1
3. Flashbacks	R	R	R	R	R	R	Factor 1
4. Emotional cue reactivity	R	R	R	R	R	R	Factor 1
5. Physical cue reactivity	R	R	R	R	R	R	Factor 1
6. Avoidance of thoughts	A	A	A	A	A	A	Factor 1
7. Avoidance of reminders	A	A	A	A	A	A	Factor 1
8. Trauma-related amnesia	NACM	D	NACM	NACM	NACM	NA	Factor 3
9. Negative beliefs	NACM	D	NACM	NACM	NACM	NA	Factor 3
10. Distorted blame	NACM	D	NACM	NACM	NACM	NA	Factor 3
11. Persistent negative emotional state	NACM	D	NACM	NACM	NACM	NA	Factor 3
12. Lack of interest	NACM	D	NACM	AN	NACM	AN	Factor 2
13. Feeling detached	NACM	D	NACM	AN	NACM	AN	Factor 2
14. Inability to experience positive emotions	NACM	D	NACM	AN	NACM	AN	Factor 2
15. Irritable/ angry	AR	D	DA	DA	EB	EB	Factor 2
16. Recklessness	AR	AR	DA	DA	EB	EB	Factor 2
17. Hypervigilance	AR	AR	AA	AA	AA	AA	Factor 1
18. Exaggerated state	AR	AR	AA	AA	AA	AA	Factor 1
19. Difficulty concentrating	AR	D	DA	DA	DA	DA	Factor 1
20. Sleep disturbance	AR	D	DA	DA	DA	DA	Factor 1

Note: *R* re-experiencing, *A* avoidance, *NACM* negative alterations in cognitions and mood, *AR* alterations in arousal and reactivity, *AN* anhedonia, *DA* dysphoric arousal, *AA* anxious arousal, *NA* negative affect, *EB* externalizing behaviour

In a number of studies, the two six factor models (Anhedonia Model: [5, 6, 8, 15, 18]); (Externalizing Model: [5, 15, 18, 19]) showed good fit with the data and outperformed all models comprising fewer factors. However, the seven-factor Hybrid Model has been found to be the best fitting model [5, 6, 8, 11, 15, 18]. In sum, the literature on the latent structure of the DSM-5 symptoms as assessed by the PCL-5 is still unclear, although the Hybrid Model has recently been supported most consistently.

Importantly, a recent study [22] examined the impact of these different psychometric models on prevalence rates and found a considerable variation of PTSD rates depending upon the latent symptom profile. This finding indicates that diagnostic implications of factor analytic modelling of the PTSD symptom structure are to be considered in future studies.

### The current study

The aims of the current study were threefold. First, we developed a German version of the PCL-5 and tested its psychometric properties, i.e. reliability, convergent validity and diagnostic utility. As evidence on the diagnostic utility of the PCL-5 is still sparse, we tested how sensitive and specific provisional diagnoses of PTSD established from the PCL-5 are when compared to the gold standard CAPS-5 diagnoses. Finally, we tested the structural validity of the German PCL-5 directly comparing the DSM-5 model of PTSD to other models suggested in the literature.

## Methods

### Procedure and participants

The investigated sample (*N* = 341) was diverse in terms of demographic characteristics and reported various types of traumatic events (see Table 2 for sample characteristics). Inclusion criteria were exposure to at least one traumatic event and at least one month elapsed since the trauma. This was assessed via self-report on the LEC. Recruitment took place in five different treatment centers specializing in the treatment of trauma-related disorders through staff describing the study to the patients and via newspaper announcement from June 2014 until December 2015. In total, *N* = 566 participants with a lifetime trauma history were informed about the study and *n* = 352 signed written consent. Most of the participants were treatment-seeking (*n* = 320), only *n* = 32 were recruited via newspaper announcement and were non-treatment seeking participants. Finally, *n* = 341 completed the assessment and were included in the analysis. Test-retest assessment was sent only to those participants who had not started treatment yet in the month following the first assessment (*n* = 80); *n* = 47 treatment-seeking participants and *n* = 31 non-treatment seeking participants completed the retest (22.2%) three weeks after the first assessment.

**Table 2:** Sample characteristics (*N* = 341)

Characteristic	*N (%)*	*M (SD)*	Range
Age		37.54 (12.16)	18 – 76
Gender^a^			
Female	192 (56.3%)		
Male	148 (43.4%)		
Marital Status^b^			
Single	189 (55.4%)		
Married	111 (32.5%)		
Divorced	33 (9.7%)		
Widowed	4 (1.2%)		
Children^c^			
Yes	176 (51.7%)		
No	156 (45.7%)		
Employment Situation^d^			
Not Working	75 (21.0%)		
Working Part-Time	34 (10.0%)		
Working Full-Time	137 (40.2%)		
Retired	42 (12.3%)		
Other	45 (13.2%)		
Most Frequent Traumatic Events^e^			
Natural disaster	49 (14.7%)		
Traffic Accident	143 (42.7%)		
Other Accident	62 (18.7%)		
Physical Assault	185 (54.9%)		
Sexual Assault	142 (42.1%)		
Combat	74 (22.4%)		
PTSD according to CAPS-5^f^			
Yes	207 (60.7%)		
No	129 (37.4%)		

Note: missing data ^a^ *n* = 1 (0.3%); ^b^ *n* = 4 (1.2%); ^c^ *n* = 9 (2.6%); ^d^ *n* = 8 (2.3%); ^e^According to the LEC; multiple entries per person were possible; ^f^ *n* = 5 (1.5%)

Participants recruited in both ways were fully informed about the purpose and procedures of the study before providing written informed consent. The assessment for both groups included a clinical interview and a questionnaire battery with a varying order of the measures. The interview was conducted either by registered clinical psychologists (two centers) or trained psychologists with at least a bachelor’s degree (three centers). Interviewers received an intensive two-day training workshop and were continuously supervised throughout the study. The study was approved by the institutional research ethics committee of the University of Münster.

### Measures

Trauma exposure was measured with the German version of the *Life Events Checklist for DSM-*5 (LEC-5; [23]), a self-report measure assessing exposure to 16 traumatic events and one additional item for any other extraordinarily stressful event. Next, participants completed the German version of the *PTSD Checklist for DSM-5* (PCL-5; [4]). The PCL-5 is a self-report measure and consists of 20 items that correspond to the DSM-5 criteria for PTSD. Participants report their intensity of symptoms over the past month on a 5-point-scale ranging from *0 = not at all* to *4 = extremely*. The translation of the LEC-5 and PCL-5 included several steps. First, the original version was translated into German. Next, the translation was back-translated into English by a professional translator, and the back-translation was compared to the original English version. Discrepancies were resolved and corrected until the German version was adequate (see Additional file 1: Appendix A for the final version of the measure).

The German version of the *Clinician Administered PTSD Scale for DSM-5* (CAPS-5) ([12]; German version: [12, 24]) was administered to determine whether participants fulfilled the diagnostic criteria for PTSD according to DSM-5 and to obtain an interviewer rating of the severity of PTSD symptoms. The CAPS-5 is a structured clinical interview assessing the presence vs. absence of DSM-5-criteria and providing a symptom severity score. Clinicians rated the frequency and intensity of each symptom over the past month on a 5-point-scale ranging from *0 = absent* to *4 = extreme/ incapacitating*.

To assess for comorbid depressive and general psychopathological symptoms, the *Beck Depression Inventory- II* (BDI-II; [25, 26]) and the *Brief Symptom Inventory* (BSI; [27, 28]) were used. Both are widely used and well-validated measures of depressive symptom severity [29] and general psychopathology [30], respectively.

### Data analysis

Analyses were conducted using SPSS 23.0 and Mplus Version 7 [31]. We first computed descriptive statistics, internal consistencies and test-retest reliabilities. Next, we evaluated the convergent validity of the PCL-5 by calculating the correlations between the PCL-5 total scores and the CAPS-5. The amount of missing data on the PCL was very low (less than 0.4% of all data points; maximum number of missing items per person was 3). In the case of missing items on the PCL, a sum score of all valid items was computed, as this is the most conservative estimate. In addition, receiver operating characteristic (ROC) curves were calculated to identify diagnostic accuracy for different cutoffs. We first tested the diagnostic utility of the cutoff 31, 32, and 33 suggested by the instrument authors and empirically identified in by Bovin and colleagues [6]. We then examined whether an alternative cutoff existed that led to higher diagnostic accuracy. We also tested the diagnostic agreement with the CAPS-5 diagnoses for the alternative scoring method using ROC. This method involves that each PCL-5 item rated at least 2 (moderately) is treated as a symptom endorsed and the DSM-5 diagnostic rule is then used to establish a provisional PTSD diagnosis. To further analyse the diagnostic utility, we calculated the sensitivity (probability that someone with a CAPS-5 diagnosis will test positive on the PCL-5), specificity (probability that someone without a CAPS-5 diagnosis will test negative on the PCL-5), the positive predictive power (probability that someone with a positive PCL-5 receives a CAPS-5 diagnosis), the negative predictive power (probability that someone with a negative PCL-5 does not receive a CAPS-5 diagnosis) and the overall efficiency (percentage of cases correctly classified). All of these analyses were conducted with SPSS 23.0.

Finally, CFA using the robust maximum likelihood procedure was conducted to evaluate six often reported structural models of PTSD (see Table 1) using Mplus. Missing data were dealt with the full information maximum likelihood (FIML) procedure. The model fit was evaluated with the comparative fit index (CFI), the Tucker-Lewis-Index (TLI), the root-mean square error of approximation (RMSEA), and the standardized root mean square residual (SRMR). A good (and adequate) fit is indicated by CFI and TLI ≥ .95 (.90- < .95), RMSEA ≤.06 (.06-.08), and SRMR ≤.08 [32]. To compare nested models, we used a chi-square difference test, for non-nested models, the Bayesian information criterion (BIC) and the Akaike information criterion (AIC) were used. A difference of 10 points represents a better fit for the model with the lower BIC value [33].

## Results

### Descriptives

Participants reported an average sum score of 39.09 (*SD* = 19.99) on the PCL-5. Sixty-three percent of the sample met or exceeded a recommended cut score of 33 for provisional PTSD diagnosis. According to the CAPS-5, 61.6% of our sample met DSM-5 criteria for PTSD. The CAPS-5 average severity score was 29.09 (*SD* = 16.42). Both symptom levels of depression as well as general psychopathology were in the moderate range (depressive symptoms: BDI-II: *M* = 22.51, *SD* = 14.35; general psychopathology: BSI: *M* = 1.28; *SD* = 0.86).

### Reliability

Internal consistency for the PCL-5 total score was high, with α = .95 for the total scale and α .79 - .89 for the subscales (see Table 3). Inter-item correlations were computed as another measure for internal consistency and ranged from .21 to .73, which can be regarded as acceptable [34] (*M* = .48; re-experiencing items: .55 - .72, avoidance items: .65, negative alterations in cognitions and mood items: .23 - .69, and alterations in arousal and reactivity items: .27 - .73).

**Table 3:** Scale-level descriptive statistics

Scale	*M*	*SD*	Possible range	Observed range	α
PCL-5	39.09	19.99	0-80	0-80	.95
PCL-5 intrusions	10.58	5.83	0-20	0-20	.89
PCL-5 avoidance	4.58	2.60	0-8	0-8	.79
PCL-5 neg. Cognitions & emotions	12.87	7.44	0-28	0-28	.86
PCL-5 hyperarousal	11.10	6.21	0-24	0-24	.84
CAPS-5	29.10	16.42	0-80	0-70	.93
BDI-II	22.51	14.35	0-63	0-55	.95
BSI	1.28	0.86	0-4	0-3.62	.97

To analyse test-retest reliability, *n* = 78 participants were re-assessed with the PCL-5 three weeks after the initial assessment. The PCL-5 total score showed a good test-retest reliability with *r*_*tt*_ = .91. A paired *t* test revealed no significant difference between both assessment times (Time 1: *M* = 28.77, *SD* = 21.13, Time 2: *M* = 26.97, *SD* = 20.86, *t*(77) = 1.78, *p* = .08). At item level, test-retest reliability ranged from .59 to .86 with a median of .74, indicating good consistency across both assessment times.

### Convergent validity and diagnostic utility

A strong correlation between the PCL-5 total score and the CAPS-5 total severity score was found (*r* = .77), indicating good convergent validity.

**Fig. 1:**
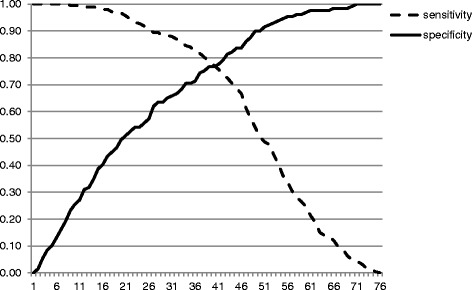
Sensitivity and Specificity curve for PCL-5 scores predicting CAPS-5 diagnoses

Receiver operating characteristic (ROC) analyses were computed with SPSS to specify sensitivity and specificity of the PCL-5 total score when compared to DSM-5 diagnoses established with the CAPS-5 (see Fig. 1). The accuracy of the PCL-5 total score was found good with the area under the curve of .85, 95% CI = [.81, .90]. When examining the cutoff of 33 recommended in the literature, diagnostic efficiency was acceptable (sensitivity = .86, specificity = .68, overall efficiency = .79). Results were very similar when applying a cutoff of 31 or 32, respectively (see Table 4). The PCL-5 symptom scoring method also led to acceptable sensitivity, specificity and overall efficiency, but performed slightly worse than the cutoff of 33. There was no other cutoff leading to a higher overall efficiency.

**Table 4:** Diagnostic utility of different PCL cutoff scores

PCL-5 Cutoff	Sensitivity	Specificity	PPP	NPP	OE	% PTSD according to this criterion
31	.88	.66	.81	.77	.79	67%
32	.87	.67	.81	.76	.79	66%
33	.86	.68	.81	.75	.79	65%
PCL-5 symptom scoring	.84	.68	.81	.73	.78	64%

*Sensitivity* probability that someone with a CAPS-5 diagnosis will test positive on the PCL-5, *Specificity* probability that someone without a CAPS-5 diagnosis will test negative on the PCL-5, *PPP* positive predictive power, *NPP* negative predictive power, *OE* overall efficiency, PCL-5 symptom scoring: each PCL-5 rated at least 2 (moderately) is treated as a symptom endorsed and the DSM-5 diagnostic rule is then used to establish a provisional PTSD diagnosis

### Structural validity

In order to test the structural validity of the German PCL-5, six different models suggested in the literature were tested (see Table 1). CFA analyses for the DSM-5 model and dysphoria model (see Table 5 for fit indices, Table 6 for factor loading and Table 7 for factor intercorrelations) showed only a moderate fit. Non-nested model comparisons yielded a slightly better model fit for the dysphoria model (RMSEA .09, 90% CI [.09 - .10], CFI .89, TLI .87, SRMR .05) than for the DSM-5 model, indicated by a ΔBIC of 14.15. For all other models (dysphoric arousal, anhedonia, externalizing behaviour, hybrid model), linear dependencies were observed in both analyses using the robust ML or the WLSMV estimator; this indicates that the models did not fit our data^3^ and could therefore not be interpreted.

**Table 5:** PCL-5 confirmatory factor analyses model results

Models	χ^2^	Df	CFI	TLI	SRMR	RMSEA	RMSEA 90%CI	BIC	AIC
DSM-5	661.61*	164	.89	.87	.049	.094	.087 - .102	20,459.19	20,206.29
Dysphoria	647.45*	164	.89	.87	.051	.093	.086 - .101	20,445.04	20,192.13

Note: *χ* ^*2*^ chi square, *df* degree of freedom, *CFI* Comparative Fit Index, *TLI* Tucker Lewis Index, *SRMR* Standardized Root Mean Square Residual, *RMSEA* Root Mean Square Error of Approximation, *BIC* Bayesian Information Criterion, *AIC* Akaike information criterion

* *p* < .001

**Table 6:** CFA standardized factor loadings

PTSD symptoms	DSM-5 model	Dysphoria model
B1. Intrusive thoughts	.85	.85
B2. Nightmares	.75	.75
B3. Flashbacks	.74	.74
B4. Emotional cue reactivity	.81	.82
B5. Physical cue reactivity	.81	.81
C1. Avoidance of thoughts	.78	.78
C2. Avoidance of reminders	.83	.83
D1. Trauma-related amnesia	.40	.40
D2. Negative beliefs	.74	.73
D3. Distorted blame	.66	.65
D4. Persistent negative emotional state	.81	.81
D5. Lack of interest	.72	.71
D6. Feeling detached	.76	.75
D7. Inability to experience positive emotions	.73	.72
E1. Irritable/ angry	.57	.58
E2. Recklessness	.47	.45
E3. Hypervigilance	.76	.82
E4. Exaggerated state	.81	.87
E5. Difficulty concentrating	.79	.78
E6. Sleep disturbance	.74	.73

**Table 7:** Correlations Among Factors for all tested models

DSM-5 model & dysphoria model				
	R	A	NACM/ D	AR
R	–	.783	.886	.922
A	.783	–	.784	.760
NACM / D	.911	.792	–	.933
AR	.849	.694	.870	–

Note: the top diagonal correlations are from the DSM-5 model, the lower diagonal correlations are from the dysphoria model

Note: *R* re-experiencing, *A* avoidance, *NACM* negative alterations in cognitions and mood, *AR* alterations in arousal and reactivity

## Discussion

The first aim of the current study was to test the psychometric properties of the German version of the PCL-5. The study was conducted on a large clinical sample with a high proportion of PTSD-positive participants. Internal consistencies and re-test reliabilities for the total scale and for all subscales were very high and comparable to those for the original PCL-5 [5, 6, 8]. In addition, we found a high correlation between the total scale and the severity rating derived from the CAPS-5, suggesting strong construct validity. Taken together, the study provides strong preliminary evidence that the German PCL-5 is a reliable and valid self-report instrument to assess PTSD symptom severity.

Although one of the aims of the PCL-5 is to enable making provisional PTSD diagnoses, to our knowledge only one prior study has directly tested the diagnostic utility of the questionnaire when compared to the gold-standard assessment using a structured clinical interview. The second aim of our study therefore was to test provisional diagnoses based on the PCL-5 against diagnoses based on the CAPS-5. Results showed that the recommended cutoff of 33 as well as the symptom scoring method both showed good diagnostic accuracy against a CAPS diagnosis however the cutoff performed slightly better. Both criteria led to high sensitivity (≥ .84), moderate specificity (≥ .66) and adequate overall efficiency (.79). It should be noted, however, that there are no universal criteria to decide what constitutes a good performance of a screening instrument (see: [35, 36]) as the relative importance of sensitivity and specificity depends on the nature of the diagnostic situation. Therefore, there may be situations where higher specificity is needed, e.g. due to reduced capacity for further assessment or treatment. As shown in Fig. 1, the PCL-5 can be accommodated to be used in these situations by choosing a higher cutoff, although it should be noted that this naturally comes at the cost of reduced sensitivity.

The final aim of our study was to test the underlying latent structure of the questionnaire. In line with earlier studies, the fit for the four-factor DSM-5 model was unsatisfactory. Most alternative models suggested in the literature could not be interpreted due to linear dependencies. An explanation for this could be the rather high occurrence of PTSD with a high diversity of trauma types and demographic characteristics in our sample. This is contrary to other studies that focused primarily on certain trauma types (e.g. military sample and veterans [5, 6]). Future studies need to investigate a possible relationship between sample characteristics and model fit.

In addition, in all tested models correlations between factors were high. Other studies also reported high factor correlations in the range of .73 - .92 [15] and .69 - .97 [10], respectively. To address the unsatisfactory fit of the tested models, a different statistical approach (e.g. network analyses) appears suitable to investigate if a model of mutually reinforcing symptoms is better to explain PTSD symptoms than the common factor models. This approach has been applied in a recent study [37] where strong connections between central PTSD symptoms (e.g. nightmares and flashbacks) has been found; the most central symptoms in this study has been negative trauma related emotions, flashbacks, detachment and physiological reactivity.

With respect to the factor structure, previous studies also led to variable results and model fit was usually not excellent. Comparing the factor structure of our data to previous studies, we find a better fit for the dysphoria model compared to the DSM-5 model (see also e.g. [15]). However, the hybrid model is usually reported as the best fitting model (see e.g.[18]). It is conceivable that the heterogeneous results regarding the factor structure of the PCL-5 specifically and the dimensional nature of PTSD more generally are at least partly due to the fact that there were significant differences in the composition of samples between studies. None of the published studies to date has used a truly representative sample, which would be a necessary next step. In addition, samples differed regarding the average PTSD symptom severity, the PTSD rate, trauma type, and demographic variables. Compared to earlier studies in the field, our sample was characterized by a particularly high PTSD rate and a predominance of treatment-seeking individuals who had suffered from civilian trauma (as compared to veteran samples in a number of earlier studies). Future research using large representative samples (i.e. samples with heterogeneous trauma types and also clinical, treatment seeking samples) is needed to provide more reliable results and formally test factorial invariance across samples. In a further step network analyses could also help to understand if the heterogeneous findings of the factor structure can be explained by varying symptom connections in different PTSD subgroups.

### Limitations

The current study shows a number of strengths, including the test of a clinical sample with a high base rate of PTSD, and the comparison of the PCL-5 with the CAPS-5. On the other hand, a number of limitations are noteworthy. Most importantly, the sample size was rather modest, which may have impacted on the CFA results. In addition, we were not able to directly compare subgroups (e.g. samples of military or veterans, samples of childhood trauma, accidental trauma). Finally, we were unable to conduct discriminant validity analyses due to the lack of appropriate instruments in our study design.

## Conclusions

Despite these limitations, the current study provides important first evidence for the German PCL-5 as a questionnaire with good reliability and high diagnostic utility. This is the first study validating the German version of the PCL-5. Results indicated that the PCL-5 is a sensitive, specific and reliable measurement for PTSD with high clinical utility. Results regarding the factor structure underlying the measure remain inconclusive as none of the models tested showed a good fit to our data. Differences to earlier findings may be due to differences in sample characteristics. Most importantly, the results underscore the need to systematically investigate the factor structure of the PCL-5 and PTSD symptoms in large representative samples.

## Additional file

Additional file 1: Appendix A. German version of the PCL-5. (DOCX 21 kb)

## Abbreviations

AIC: Akaike information criterion
BDI: Beck’s Depression Inventory
BIC: Bayesian Information Criterion
BSI: Brief Symptom Inventory
CAPS: Clinician Administered PTSD Scale
CFA: confirmatory factor analyses
CFI: Comparative Fit Index
CI: Confidence Interval
DSM: Diagnostic and Statistical Manual of Mental Disorders
EFA: exploratory factor analyses
FIML: full information maximum likelihood
M: mean
PCL: posttraumatic stress disorder checklist
PTSD: posttraumatic stress disorder
RMSEA: Root Mean Square Error of Approximation
ROC: receiver operating curve
SD: standard deviation
SRMS: standardized root mean square residual
TLI: Tucker Lewis Index

## Footnotes

1
http://www.ptsd.va.gov/professional/assessment/adult-sr/ptsd-checklist.asp, retrieved on 12 August 2016

2 Note, however, that three studies [16, 18, 19] were based on the same population.

3 The linear dependency also remained when items were recoded into binary items.
