# Supplementary material for: The German version of the Posttraumatic Stress Disorder Checklist for DSM-5 (PCL-5): psychometric properties and diagnostic utility
Source: BMC Psychiatry. 2017 Nov 28;17:379. doi: 10.1186/s12888-017-1541-6 (PMC5704375; doi:10.1186/s12888-017-1541-6)
Supplement: Additional file 1: Appendix A. — German version of the PCL-5. (DOCX 21 kb) [file 12888_2017_1541_MOESM1_ESM.docx]

**Appendix A**

*– German version of the PCL-5 -*

PCL-5

Instruktion: Nachfolgend sind Probleme aufgelistet, die Menschen manchmal als Reaktion auf ein sehr belastendes Erlebnis haben. Bitte lesen Sie jedes Problem sorgfältig und markieren Sie dann eine der Zahlen auf der rechten Seite um anzugeben, wie stark Sie im letzten Monat durch dieses Problem belastet waren.

| Im letzten Monat, wie sehr waren Sie belastet durch: | überhaupt  nicht | ein wenig | ziemlich | stark | sehr stark |
| --- | --- | --- | --- | --- | --- |
| 1. Wiederholte, beunruhigende und ungewollte Erinnerungen an das belastende Erlebnis? | 0 | 1 | 2 | 3 | 4 |
| 1. Wiederholte, beunruhigende Träume von dem belastenden Erlebnis? | 0 | 1 | 2 | 3 | 4 |
| 1. Sich plötzlich fühlen oder sich verhalten, als ob das belastende Erlebnis tatsächlich wieder stattfinden würde (als ob Sie tatsächlich wieder dort wären und es wiedererleben würden)? | 0 | 1 | 2 | 3 | 4 |
| 1. Sich emotional sehr belastet fühlen, wenn Sie etwas an das Erlebnis erinnert hat? | 0 | 1 | 2 | 3 | 4 |
| 1. Starke körperliche Reaktionen haben, wenn Sie etwas an das belastende Erlebnis erinnert hat (z.B. Herzklopfen, Schwierigkeiten beim Atmen, schwitzen)? | 0 | 1 | 2 | 3 | 4 |
| 1. Vermeidung von Erinnerungen, Gedanken oder Gefühlen in Bezug auf das belastende Erlebnis? | 0 | 1 | 2 | 3 | 4 |
| 1. Vermeidung äußerer Auslöser für Erinnerungen an das belastende Erlebnis (z.B. Personen, Plätze, Gespräche, Aktivitäten, Gegenstände oder Situationen)? | 0 | 1 | 2 | 3 | 4 |
| 1. Schwierigkeiten, sich an wichtige Teile des belastenden Erlebnisses zu erinnern? | 0 | 1 | 2 | 3 | 4 |
| 1. Starke negative Überzeugungen über sich selbst, andere Menschen oder die Welt haben (z.B. Gedanken haben wie: Ich bin schlecht, mit mir stimmt ernsthaft etwas nicht, man kann niemandem vertrauen, die Welt ist absolut gefährlich)? | 0 | 1 | 2 | 3 | 4 |
| 1. Sich selbst oder jemand anderem Vorwürfe machen in Bezug auf das belastende Erlebnis oder was danach passiert ist? | 0 | 1 | 2 | 3 | 4 |
| 1. Starke negative Gefühle haben, wie zum Beispiel Angst, Schrecken, Ärger, Schuld oder Scham? | 0 | 1 | 2 | 3 | 4 |
| 1. Verlust von Interesse an Aktivitäten, die Ihnen früher Spaß gemacht haben? | 0 | 1 | 2 | 3 | 4 |
| 1. Sich von anderen Menschen entfernt oder wie abgeschnitten fühlen? | 0 | 1 | 2 | 3 | 4 |
| 1. Schwierigkeiten, positive Gefühle zu erleben (z.B. keine Freude empfinden können oder keine liebevollen Gefühle haben können gegenüber Menschen, die Ihnen nahestehen)? | 0 | 1 | 2 | 3 | 4 |
| 1. Reizbares Verhalten, Wutausbrüche oder aggressives Verhalten? | 0 | 1 | 2 | 3 | 4 |
| 1. Zu viele Risiken eingehen oder Dinge tun, die Ihnen Schaden zufügen könnten? | 0 | 1 | 2 | 3 | 4 |
| 1. In erhöhter Alarmbereitschaft, wachsam oder auf der Hut sein? | 0 | 1 | 2 | 3 | 4 |
| 1. Sich nervös oder schreckhaft fühlen? | 0 | 1 | 2 | 3 | 4 |
| 1. Konzentrationsschwierigkeiten haben? | 0 | 1 | 2 | 3 | 4 |
| 1. Schwierigkeiten, ein- oder durchzuschlafen? | 0 | 1 | 2 | 3 | 4 |

**Appendix B**

*Table*. *Item-Level Descriptive Statistics for PTSD Checklist – 5*

| *Item* | *M* | *SE* | *SD* | *Skew* | *Kurt* |
| --- | --- | --- | --- | --- | --- |
| 1. Intrusive thoughts | 2.38 | .07 | 1.34 | -.31 | -1.17 |
| 2. Nightmares | 1.73 | .08 | 1.48 | .26 | -1.33 |
| 3. Flashbacks | 1.59 | .08 | 1.40 | .35 | -1.16 |
| 4. Emotional cue reactivity | 2.68 | .07 | 1.32 | -.69 | -.68 |
| 5. Physical cue reactivity | 2.23 | .08 | 1.45 | -.27 | -1.30 |
| 6. Avoidance of thoughts | 2.36 | .08 | 1.40 | -.45 | -1.07 |
| 7. Avoidance of reminders | 2.24 | .08 | 1.47 | -.27 | -1.29 |
| 8. Trauma-related amnesia | 1.33 | .08 | 1.42 | .71 | -.88 |
| 9. Negative beliefs | 1.87 | .08 | 1.53 | .11 | -1.49 |
| 10. Distorted blame | 1.84 | .08 | 1.48 | .16 | -1.39 |
| 11. Persistent negative emotional state | 2.39 | .08 | 1.43 | -.36 | -1.25 |
| 12. Lack of interest | 1.92 | .08 | 1.38 | .06 | -1.26 |
| 13. Feeling detached | 1.86 | .08 | 1.43 | .06 | -1.35 |
| 14. Inability to experience positive emotions | 1.70 | .08 | 1.43 | .26 | -1.27 |
| 15. Irritable/ angry | 1.62 | .08 | 1.39 | .36 | -1.15 |
| 16. Recklessness | .93 | .06 | 1.17 | 1.09 | .15 |
| 17. Hypervigilance | 2.09 | .08 | 1.39 | -.11 | -1.24 |
| 18. Exaggerated state | 1.98 | .08 | 1.41 | -.02 | -1.31 |
| 19. Difficulty concentrating | 2.19 | .08 | 1.45 | -.26 | -1.32 |
| 20. Sleep disturbance | 2.31 | .08 | 1.54 | -.28 | -1.45 |

*Note*. *n*s ranged from 338-341. PCL-5 = PTSD Checklist – 5. All PCL-5 item scores ranged from 0-4. Parameter estimates are reported for Skewness and Kurtosis
